# CT-based skeletal muscle loss for predicting poor survival in patients with hepatocellular carcinoma experiencing curative hepatectomy plus adjuvant transarterial chemoembolization: a preliminary retrospective study

**DOI:** 10.1186/s40001-022-00760-6

**Published:** 2022-07-25

**Authors:** Siwei Yang, Zhiyuan Zhang, Tianhao Su, Jianan Yu, Shasha Cao, Haochen Wang, Long Jin

**Affiliations:** Department of Interventional Radiology, Beijing Friendship Hospital, Capital Medical University, No. 95 Yongan Rd., Beijing, 100050 China

**Keywords:** Skeletal muscle index, Hepatocellular carcinoma, Curative hepatectomy, Adjuvant transarterial chemoembolization, Prognosis

## Abstract

**Background:**

To evaluate the prognostic value of skeletal muscle index (SMI) and its change in patients with hepatocellular carcinoma (HCC) experiencing curative hepatectomy plus adjuvant transarterial chemoembolization (TACE).

**Materials and methods:**

A total of 62 patients with HCC who underwent adjuvant TACE after curative hepatectomy were analysed retrospectively. Skeletal muscle area at the third lumbar level was quantitated using computed tomography images and was normalized for height squared to obtain skeletal muscle index (SMI). Skeletal muscle loss (SML) over 6 months was computed with two SMIs before and after hepatectomy plus adjuvant TACE. Correlation analyses were preformed to investigate factors associated with SML. The curves of cause-specific survival (CSS) were analysed using the Kaplan–Meier method. A Cox proportional hazards model was used to assess prognostic factors.

**Results:**

Low SMI was diagnosed in 23(37.1%) patients preoperatively. The median SML standardized by 6 months was − 1.6% in the entire cohort. Liver cirrhosis and microvascular invasion correlated negatively with SML, respectively (r = − 0.320, *P* = 0.002; r = − 0.243, *P* = 0.021). Higher SML (< − 2.42%) predicted a significant reduction in CSS (*P* = 0.001), whereas low SMI did not(*P* = 0.687). Following the multivariate analysis for CSS, AFP > 400 ng/ml (HR, 5.643; 95%CI, 3.608–17.833; *P* < 0.001) and SML < − 2.42%(HR, 6.586; 95%CI, 3.610–22.210; *P* < 0.001) were independent predictors for poor CSS.

**Conclusions:**

Skeletal muscle loss during hepatectomy plus adjuvant TACE was remarkable. Higher SML was an independent risk factor for CSS in patients with HCC, especially those with liver cirrhosis.

## Introduction

Malnutrition is usually observed in patients with chronic liver disease (CLD) and is caused by an imbalance of protein synthesis and breakdown resulting from abnormal liver synthesis, metabolism, detoxification and immune function [1]. However, due to fluid overload or obesity, malnutrition risk evaluation is often overlooked. In fact, malnutrition has recently been highlighted in clinical practice for CLD patients. As a new entity in the International Classification of Diseases, sarcopenia evaluated by low skeletal muscle index (SMI) at radiologic images and grip strength loss has become of increasing importance in clinical management and survival assessment for patients with hepatocellular carcinoma (HCC) [2, 3].

Currently, only performance status and serum albumin contribute to Child–Pugh and MELD scoring as indicators of nutritional condition for HCC patients with CLD, especially in the context of cirrhosis. Since patients with resectable HCC in the real world prone to be classified as having no or low risk owing to well liver function reserve, the differentiation value of some malnutrition screening tools, such as the NRS-2002 or royal-free hospital nutritional prioritization tool, is limited.

SMI measured by radiologic images reflecting skeletal muscle mass has superior accessibility and repeatability in evaluating the quantity of skeletal muscle for HCC patients [4]. Computed tomography (CT) and magnetic resonance imaging were routinely used in the diagnosis of HCC and tumor surveillance in follow-up. The latest review summarized that low SMI or sarcopenia was associated with a poor prognosis for HCC patients in different tumor stages who received liver resection or systemic therapy [5]. As noted, the HCC patients at early stage tend to have better liver function reserve, lower tumor burden and nonsignificant weight loss during treatment, and they recovered their weight quickly after diet adjustment. Therefore, the occurrence of sarcopenia and the loss of skeletal muscle mass during treatment in such patients may be overlooked extensively.

In the light of positive impact of postoperative adjuvant transarterial chemoembolization (TACE) on prognosis of HCC patients received curative hepatectomy, which was validated in two randomized controlled trials [6, 7], this combined therapy was recommended for the HCC patients at high risk of recurrence in the latest guidelines of primary liver cancer in China [8]. However, Kobayashi, A.et al. [9] showed the skeletal muscle mass is depleted significantly after hepatectomy, and repetitive platinum-based chemotherapy may also yield a negative impact [10–12]. To date, little is known about the prognostic value of SMI or related measurements for patients with HCC experiencing curative hepatectomy plus adjuvant TACE. Herein, this study retrospectively investigated whether the stratification of these HCC patents using skeletal muscle index or related parameters could identify subgroups with different survival risks.

## Methods and methods

### Patients selection

**Fig. 1:**
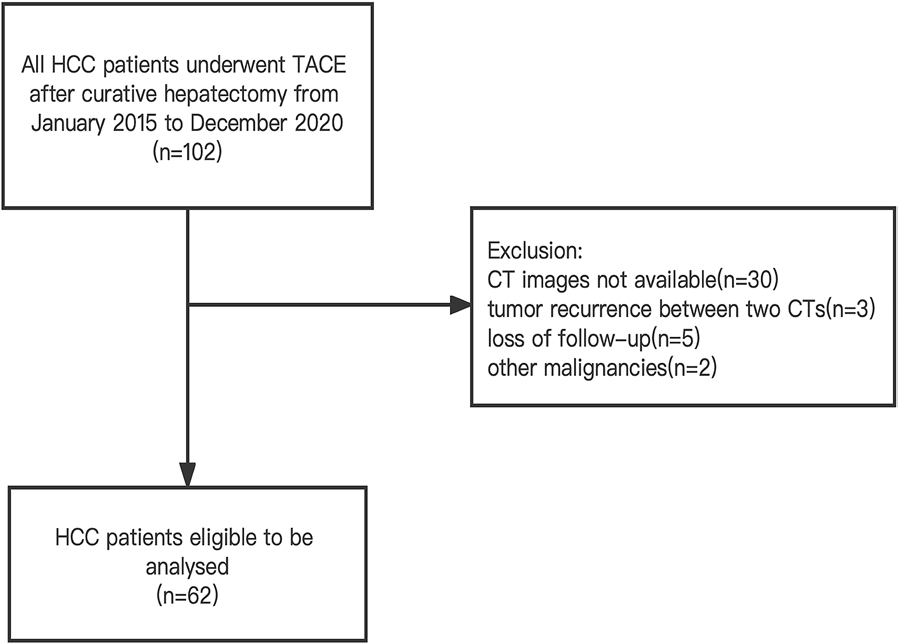
Flowchat shows patient selection

Patients with HCC who underwent adjuvant TACE after curative hepatectomy from January 2015 to December 2020 were consecutively and retrospectively analysed. The presence of at least one high risk characteristic including maximum tumor diameter > 5 cm, tumor microvascular invasion (MVI), multiple tumors and liver cirrhosis was indication to preform adjuvant TACE since 2014 in our institution. Inclusion criteria were as follows: ①curative hepatectomy preformed as initial treatment and HCC diagnosed pathologically; ②the presence of at least one high risk characteristic of poor prognosis; ③postoperative adjuvant TACE conducted at least once. Exclusion criteria were as follows: ①abdominal CT images not available within 2 weeks before and 5–8 months after hepatectomy; ②tumor recurrence found between two CT examinations; ③a history of other malignancies; ④patients with missing data or loss of follow-up. The flowchart of this study is shown in Fig. 1. The study procedures conformed to the ethical guidelines of the Declaration of Helsinki, and the institutional review board approved this retrospective study. The requirement for informed consent of recruitment was waived.

### Hepatectomy and postoperative adjuvant TACE

Patients with Barcelona Clinic Liver Cancer Group (BCLC) 0 or A and selective BCLC B stage HCC were candidates for curative hepatectomy referring to all tumor lesions found by preoperative imaging and intraoperative exploration. R0 resection (negative tumor involvement of margin in specimen) was confirmed histologically. Patients with BCLC B stage HCC, whose lesions were located at the same segment or lobe of liver and residual liver volume was sufficient, were considered to be eligible for surgery.

The first TACE was performed 3–4 weeks after hepatectomy. According to the laboratory results and performance status, TACE was conducted again after 4 weeks. Considering that Chinese guideline recommendation and protection of liver function reserve, no more than 3 times TACE sessions were conducted. Hepatic angiography was performed to clarify the liver feeding artery. Chemoemboliztion, including infusion of 50 mg/m^2^ oxaliplatin, 250 mg/m^2^ fluorouracil, 100 mg/m^2^ calcium leucovorin and embolization of 2–5 ml iodized oil, was performed through the left and right hepatic arteries unselectively.

### SMI Quantitation

**Fig. 2:**
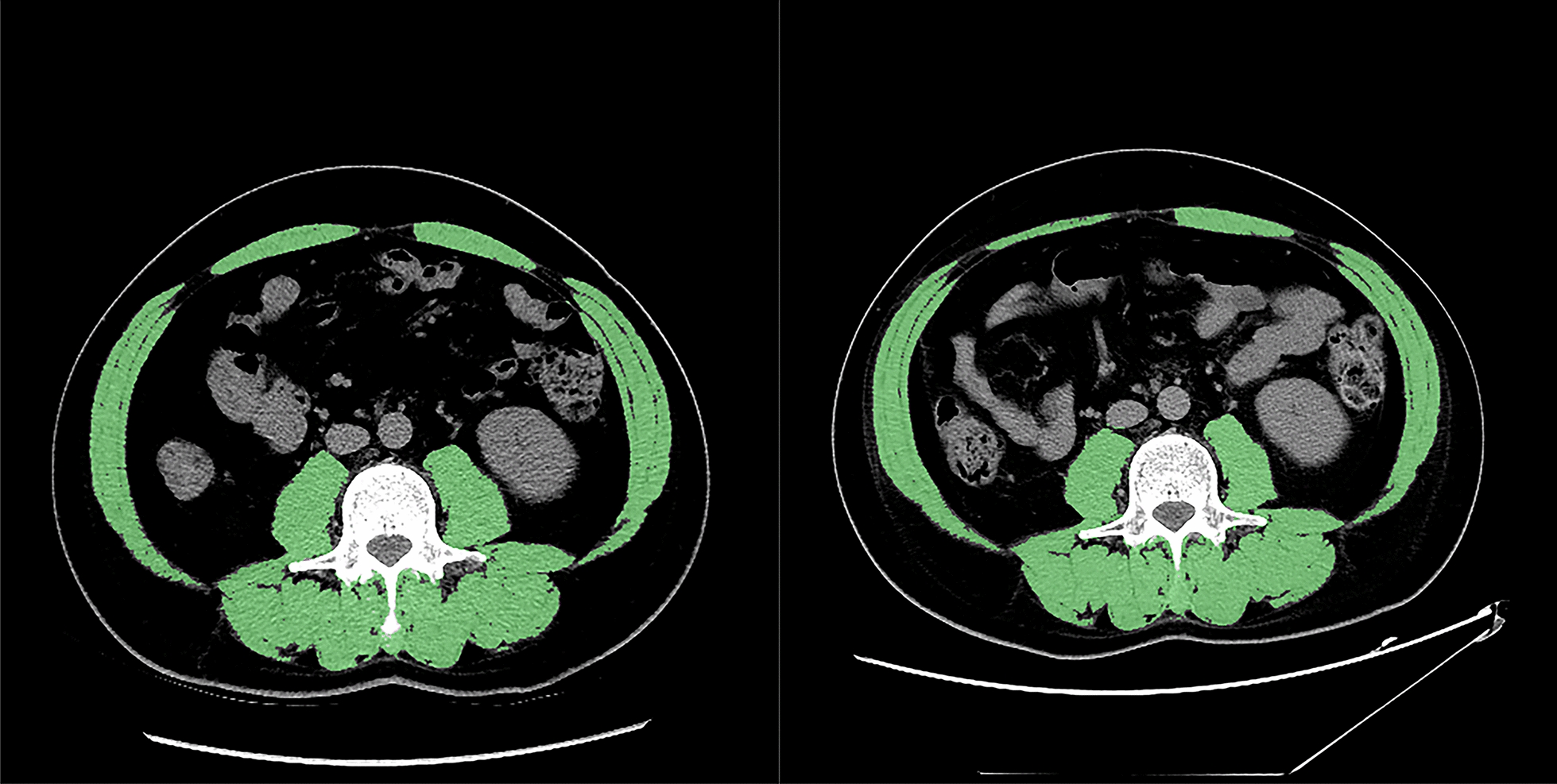
Skeletal muscle loss marked by green mask over 171 days in a 52-year-old man with a single moderate differentiated HCC lesion. **a** Preoperative skeletal muscle index (SMI) on axial CT image at the L3 level was 46.97 cm^2^/m^2^. **b** Postoperative SMI at the same plane on CT image was 40.82 cm^2^/m^2^

Mimics Research software (Version 19.0, Materialise NV, Leuven, Belgium) was applied to calculate the targeted skeletal muscle area on preoperative and postoperative CT cross-sectional images at the level of the third lumbar vertebra level (L3), including psoas major, erector spinae, quadratus lumborum, transverse abdominis, internal and external oblique and rectus abdominis. The standard Hounsfield units-derived measurement on CT image as reference methods for quantifying skeletal muscle was proposed by Mitsiopoulos, N. et al [13]. Accordingly, the CT images were segmented by density thresholds ranging from − 29 to 150 Hounsfield units, and boundaries of the targeted area were manually adjusted as needed (Fig. 2). SMI was derived from the formula: skeletal muscle area(cm^2^)/height^2^(m^2^).

### Definitions and follow-ups

Liver cirrhosis was confirmed by histopathology of the liver parenchyma adjacent to the tumor. Major and minor hepatectomy were defined as the resection of ≥ 1 or < 1 hepatic segments, respectively. The adverse events were categorized as minor and major classifications according to whether the presence of additional therapy, prolonged hospitalization and severe consequences or not [14]. The criteria of low SMI were 42 cm^2^/m^2^ for men and 38 cm^2^/m^2^ for women recommended by the working group for creation of sarcopenia assessment criteria of Japan Society of Hepatology [15]. Obesity was defined as body mass index(BMI) over 27.5 kg/m^2^ according to the World Health Organization criteria for obesity in Asian population [16]. Multiphase CT and laboratory tests were performed 1 month after resection, every 3 months thereafter. The quantitative assessment of SMI change was obtained using the following formula: SML = [(postoperative SMI − preoperative SMI) × 180/interval days between CT examinations] /preoperative SMI × 100%. The endpoint was cause-specific survival (CSS), defined as the interval between hepatectomy and the date of the last follow-up (30th April, 2021) or death. The cause of death was HCC or liver disease-related events (i.e., liver failure, variceal bleeding or hepatic encephalopathy).

### Statistical analysis

Continuous variables expressed as the mean ± standard deviation or median (interquartile range) were compared by two-sample *t* tests or Mann–Whitney *U* tests, and categorical variables expressed as number (percentage) were compared by *χ*^2^ test or Fisher’s exact tests. Correlation analyses were performed using the Spearman or Kendall’s tau-b analysis. The optimal cutoff value of SML was obtained by the receiver operating characteristic (ROC) curve according to the Youden index related to the endpoints. Survival curves were generated using the Kaplan–Meier method and analysed with the log-rank test. A Cox proportional hazard model was used to analyse prognostic factors of CSS. Univariable analyses were performed after proportional hazard verification, and variables with *P* < 0.1 were included in multivariate analysis. *P* < 0.05 was considered statistically significant. All statistical analyses were performed using SPSS statistics software (Version 26.0; IBM Corp., Armonk, NY) and R software (version 4.0.3.; http:// www.r-project.org/).

## Results

### Patient demographics

A total of 62 HCC patients were eligible to be reviewed in this study. The baseline demographic and clinical characteristics of all patients are listed in Tables 1 and 3 in Appendix according to different SML groups and SMI groups, respectively. The optimal cutoff value of SML was − 2.42%, as determined by ROC curve analysis. Compared with the lower SML group, patients in the higher SML group (SML < − 2.42%) had a higher frequency of liver cirrhosis (*P* = 0.005), tumor MVI(*P* = 0.048) and neutrophil to lymphocyte ratio (NLR) level(*P* = 0.026), but fewer TACE sessions(*P* = 0.031). In addition, male patients with a higher baseline SMI had more skeletal muscle loss(*P* = 0.021). In the entire cohort, liver cirrhosis was identified in 36(58.1%) patients. Low SMI was diagnosed in 23(37.1%) patients preoperatively. The median time between two CT examinations was 188(164–218) days. A total of 164 adjuvant TACE sessions were administrated and all adverse events related to interventional procedures were nonsignificant, mainly including embolism syndrome and mild decreasing appetite. Baseline Child–Pugh A class was observed in all participants, and there was no deterioration of Child–Pugh class after combined treatment.

**Table 1:** Baseline demographic and clinical characteristics in higher and lower SML groups

Variable	Total (n = 62)	Higher SML (n = 22)	Lower SML (n = 40)	*P* value
Sex (male)	49(79.0)	17(77.3)	32(80.0)	0.801
Age (year)	59.39 ± 10.6	57.3 ± 11.2	60.5 ± 10.2	0.258
BMI (kg/m^2^)				
Male	24.1 ± 3.4	24.8 ± 3.2	23.7 ± 3.5	0.291
Female	22.5 ± 3.2	21.8 ± 1.7	23.0 ± 3.9	0.557
SMI (kg^2^/m^2^)				
Male	45.0 ± 6.9	48.0 ± 8.1	43.3 ± 5.6	0.021
Female	39.0 ± 5.5	39.5 ± 8.4	38.6 ± 3.2	0.820
SML (%)				
Male	− 1.5 ± 3.2	− 4.9 ± 1.5	0.38 ± 2.1	< 0.001
Female	− 1.4 ± 3.2	− 4.9 ± 1.3	0.60 ± 1.8	0.002
Aetiology (HBV/HCV/others)	49(79.0)/3(4.8)/10(16.1)	19(86.4)/0/3(13.6)	30(75.0)/3(7.5)/7(17.5)	0.473
diabetes mellitus	16(25.8%)	8(36.4%)	8(20.0%)	0.226
BCLC (0/A/B)	6(9.7)/53(85.5)/3(4.8)	4(18.2)/16(72.7)/2(9.1)	2(5.0)/37(92.5)/1(2.5)	0.109
Tumor number (solitary/multiple)	54(87.1)/8(12.9)	19(86.4)/3(13.6)	35(87.5)/5(12.5)	0.898
Maximum tumor diameter (cm)	4.3 ± 2.1	3.9 ± 2.3	4.5 ± 1.9	0.278
Differentiation(well or moderate/poor)	54(87.1)/8(12.9)	18(81.8)/4(18.2)	36(90.0)/4(10.0)	0.438
Hepatectomy (major/minor)	49(79.0)/13(21.0)	18(81.8)/4(18.2)	31(77.5)/9(22.5)	0.756
MVI (presence/absence)	13(21.0)/49(79.0)	8(36.4)/14(63.6)	5(12.5)/35(87.5)	0.048
Cirrhosis (presence/absence)	36(58.1)/26(41.9)	18(81.8)/4(18.2)	18(45.0)/22(55.0)	0.005
TACE sessions (1/2/3)	5(8.1)/12(19.4)/45(72.6)	1(4.5)/8(36.4)/13(59.1)	4(10.0)/4(10.0)/32(80.0)	0.031
Child–Pugh class (A/B)	62(100.0)	22(100.0)/0	40(100.0)/0	-
MELD-Na score	7.0(6.0 ~ 9.0)	7.0(6.0 ~ 8.0)	7.0(6.0 ~ 9.0)	0.359
ALB (g/L)	40.0 ± 3.8	39.8 ± 4.2	39.7 ± 3.6	0.878
TBIL (umol/L)	15.5(11.9 ~ 22.6)	14.3(11.6 ~ 17.0)	16.8(13.1 ~ 24.7)	0.064
ALT (U/L)	23.0(17.0 ~ 31.3)	24.5(15.0 ~ 30.3)	22.0(18.0 ~ 39.3)	0.466
AST (U/L)	29.2(22.6 ~ 36.6)	26.5(21.0 ~ 32.5)	30.7(23.1 ~ 36.9)	0.406
PLT (10^9^/L)	159.4 ± 59.4	153.6 ± 62.8	162.3 ± 58.0	0.573
AFP (ng/ml)	20.7(2.7 ~ 305.9)	53.6(2.6 ~ 325.4)	15.0(2.8 ~ 297.9)	0.953
NLR	1.9 ± 1.0	2.3 ± 1.1	1.7 ± 0.9	0.026

**Table 3:** Baseline demographic and clinical characteristics in high and low SMI group

variable	Low SMI (n = 23)	high SMI (n = 39)	*P* value
Sex (male)	18(78.3)	31 (79.5)	1.000
Age (year)	61.0 ± 11.6	57.9 ± 9.8	0.159
BMI (kg/m^2^)			
Male	21.7 ± 2.2	25.5 ± 3.2	< 0.001
Female	23.0 ± 3.2	22.2 ± 3.3	0.686
SMI (kg^2^/m^2^)			
Male	38.2 ± 3.3	48.8 ± 5.2	< 0.001
Female	33.9 ± 2.7	42.1 ± 4.2	0.003
SML (%)			
Male	− 0.4 ± 3.0	− 2.1 ± 3.1	0.071
Female	− 2.3 ± 2.9	− 1.0 ± 3.4	0.661
Aetiology (HBV/HCV/others)	13 (56.5)/3 (13.0)/7 (30.4)	36 (92.3)/0/3 (7.7)	0.001
BCLC (0/A/B)	1 (4.3)/20 (87.0)/2 (8.7)	5 (12.8)/33 (84.6)/1(2.6)	0.329
Tumor number (solitary/multiple)	19 (82.6)/4 (17.4)	35 (89.7)/4(10.3)	0.454
Maximum tumor diameter (cm)	4.8 ± 2.5	4.0 ± 1.8	0.115
Differentiation(well or moderate/poor)	21 (91.3)/2 (8.7)	33 (84.6)/6 (15.4)	0.698
Hepatectomy (major/minor)	20 (87.0)/3 (13.0)	29 (74.4)/10 (25.6)	0.338
MVI (presence/absence)	5 (21.7)/18 (78.3)	8 (20.5)/31 (79.5)	1.000
Cirrhosis (presence/absence)	8 (34.8)/15 (65.2)	28 (71.8)/11 (28.2)	0.007
TACE sessions (1/2/3)	3 (13.0)/4 (17.4)/16 (69.6)	2 (5.1)/8 (20.5)/29 (74.4)	0.615
Child–Pugh class (A/B)	23 (100.0)/0	39 (100.0)/0	-
MELD-Na score	7.0 (6.0 ~ 9.0)	7.0 (6.0 ~ 9.0)	0.372
ALB (g/L)	38.8 ± 3.3	40.2 ± 4.0	0.168
TBIL (umol/L)	14.4 (11.7 ~ 18.5)	15.9 (11.9 ~ 22.6)	0.590
ALT (U/L)	21.0 (16.0 ~ 31.0)	25.0 (18.0 ~ 37.0)	0.166
AST (U/L)	27.2 (20.2 ~ 36.1)	30.2 (23.2 ~ 37.0)	0.175
PLT (10^9^/L)	170.2 ± 66.7	153.1 ± 54.6	0.278
AFP (ng/ml)	20.2 (2.9 ~ 414.9)	21.3 (2.6 ~ 152.3)	0.498
NLR	1.9 ± 0.9	2.0 ± 1.0	0.710

**Table 2:** Univariate and multivariate analysis using Cox proportional hazards model for cause-specific survival

Variables	Univariate analysis		Multivariate analysis	
	HR(95%CI)	*P* value	HR(95%CI)	*P value*
Age (> 60y)	0.384 (0.122 ~ 1.202)	0.288		
Sex (male)	2.053 (0.453 ~ 9.308)	0.351		
SML (< − 2.42%)	5.761 (1.761 ~ 18.848)	0.004	6.586 (3.610 ~ 22.210)	< 0.001
BMI (> 23 kg/m^2^)	3.316 (1.018 ~ 10.802)	0.147		
Maximum tumor diameter (> 5 cm)	1.429 (0.466 ~ 4.384)	0.532		
tumor number (multiple)	1.871 (0.513 ~ 6.826)	0.343		
MVI	1.163 (0.319 ~ 4.238)	0.819		
Cirrhosis	2.158 (0.593 ~ 7.845)	0.243		
BCLC (B)	2.582 (0.571 ~ 11.688)	0.218		
TBIL (> 17.1ummol/L)	0.724 (0.233 ~ 2.355)	0.591		
ALB (< 35 g/L)	3.333 (0.730 ~ 15.212)	0.120		
ALT (> 40 U/L)	0.330 (0.043 ~ 2.541)	0.287		
PLT (< 100 × 10^9^/L)	2.364 (0.648 ~ 8.626)	0.193		
AFP (> 400 ng/ml)	4.478 (1.494 ~ 13.419)	0.007	5.643 (3.608 ~ 17.833)	< 0.001
TACE (> 2 sessions)	0.561 (0.172 ~ 1.822)	0.336		
NLR (> 1.83*)	1.127 (0.378 ~ 3.364)	0.830		

^*^Median value;

### The impacts of SMI and SML on CSS

**Fig. 3:**
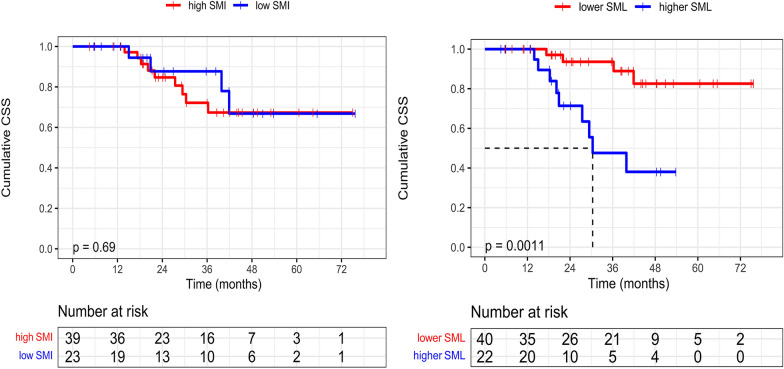
Comparisons of Survival curves in different SMI or SML subgroups. **a** Cumulative CSS curves between the baseline low and high SMI groups. **b** Cumulative CSS curves between the lower and higher SML groups

The cumulative CSS at 1, 3 and 5 years was 100.0%, 85.8% and 67.0%, respectively. Baseline SMI based on Japanese criteria was unable to stratify the liver-related mortality risk of patients (*P* = 0.687, Fig. 3a).

The follow-up times in lower and higher SML groups were 36.1(19.2–45.1) months and 21.6(17.4–32.8) months, respectively. During the follow-up, Four(10.0%) out of the 40 patients in the lower SML group died, and the cumulative CSS at 1, 3 and 5 years was 100%, 93.6% and 82.6%, respectively. Nine(40.9%) out of the 22 patients in the higher SML group died, and the cumulative CSS at 1, 3 and 5 years was 100%, 71.4% and 38.1%, respectively. The difference in CSSs between the two groups was statistically significant (median CSS: NA vs. 911.0 days, *P* = 0.001, Fig. 3b).

### The impact of obesity on CSS

**Fig. 4:**
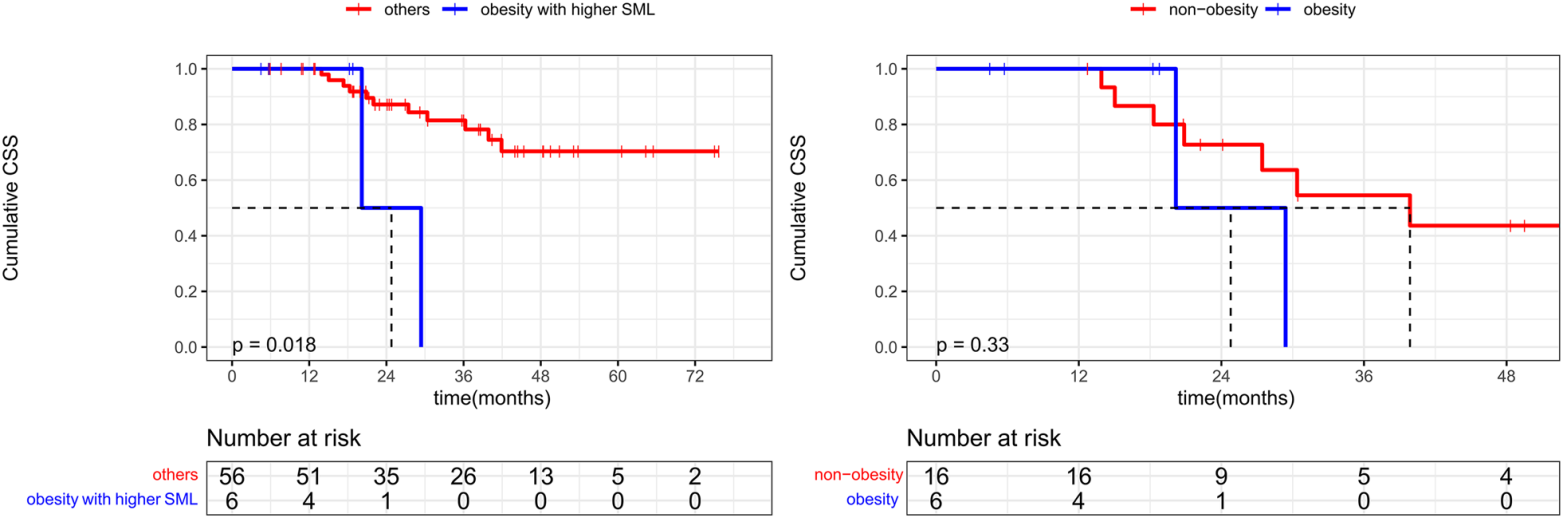
**a** Obesity patients with higher SML had a reduced CSS compared with other patients. **b** CSS was trending toward but not significantly different between obesity and non-obesity patients in the higher SML group(median CSS: 605.0 days vs. 1196.0 days)

Of twelve obese patients in this study, there were six obese patients with higher SML, but no individual was categorized as low SMI obesity. CSS did not differ significantly between obese and non-obese patients (*P* = 0.734). After adjustment for stratification of SML, a significant separation of CSS curves between obese patients with higher SML and residual patients (median CSS: 605.0 days vs. NA, *P* = 0.018, Fig. 4a). The difference between obese and non-obese patients in the higher SML group showed a clear trend but did not reach statistical significance(median CSS: 605.0 days vs. 1196.0 days, *P* = 0.330, Fig. 4b).

### Features of SML between patient subgroups

**Fig. 5:**
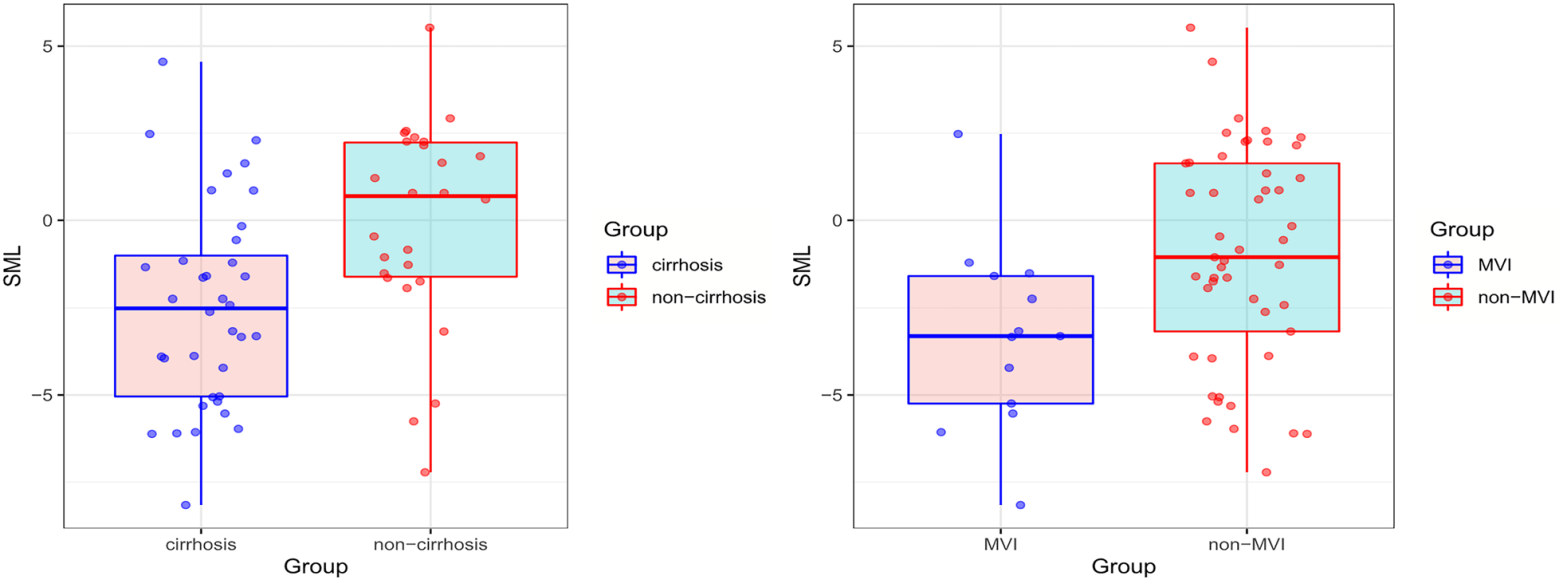
**a** Boxplot diagram showed patients with cirrhosis had higher SML than those without cirrhosis (median SML: − 2.52% vs. 0.69%, *P* = 0.003). **b** Higher SML was observed in patients with MVI than those without MVI (median SML:  − 3.32% vs. − 0.90%, *P* = 0.016)

The median SML standardized by 6 months was − 1.6% in the entire cohort. Correlation analyses were used to further clarify the potential relationship between SML and other characteristics, including clinical variables (sex, age, BMI, BCLC class, tumor number, maximum tumor diameter, tumor differentiation, MVI, cirrhosis and TACE sessions) as well as routine laboratory values (TBIL, ALT, AST, PLT, AFP and NLR). Only two factors, the presence of cirrhosis (*r* = − 0.320, *P* = 0.002) and MVI (*r* = − 0.243, *P* = 0.021), were negatively correlated with SML. Besides, patients with cirrhosis had a higher SML than those without cirrhosis (median SML: − 2.52% vs. 0.69%, *P* = 0.003, Fig. 5a). Likewise, a higher SML was seen in the patients with MVI than those without MVI (median SML: − 3.32% vs. − 0.90%, *P* = 0.016, Fig. 5b). When the cohort was divided into different subgroups according to NLR level categorized by median value or TACE sessions (> 2), there was no significant differences in SML between different subgroups, respectively (*P* = 0.746; *P* = 0.081).

### Association between lower SML and poor CSS

**Fig. 6:**
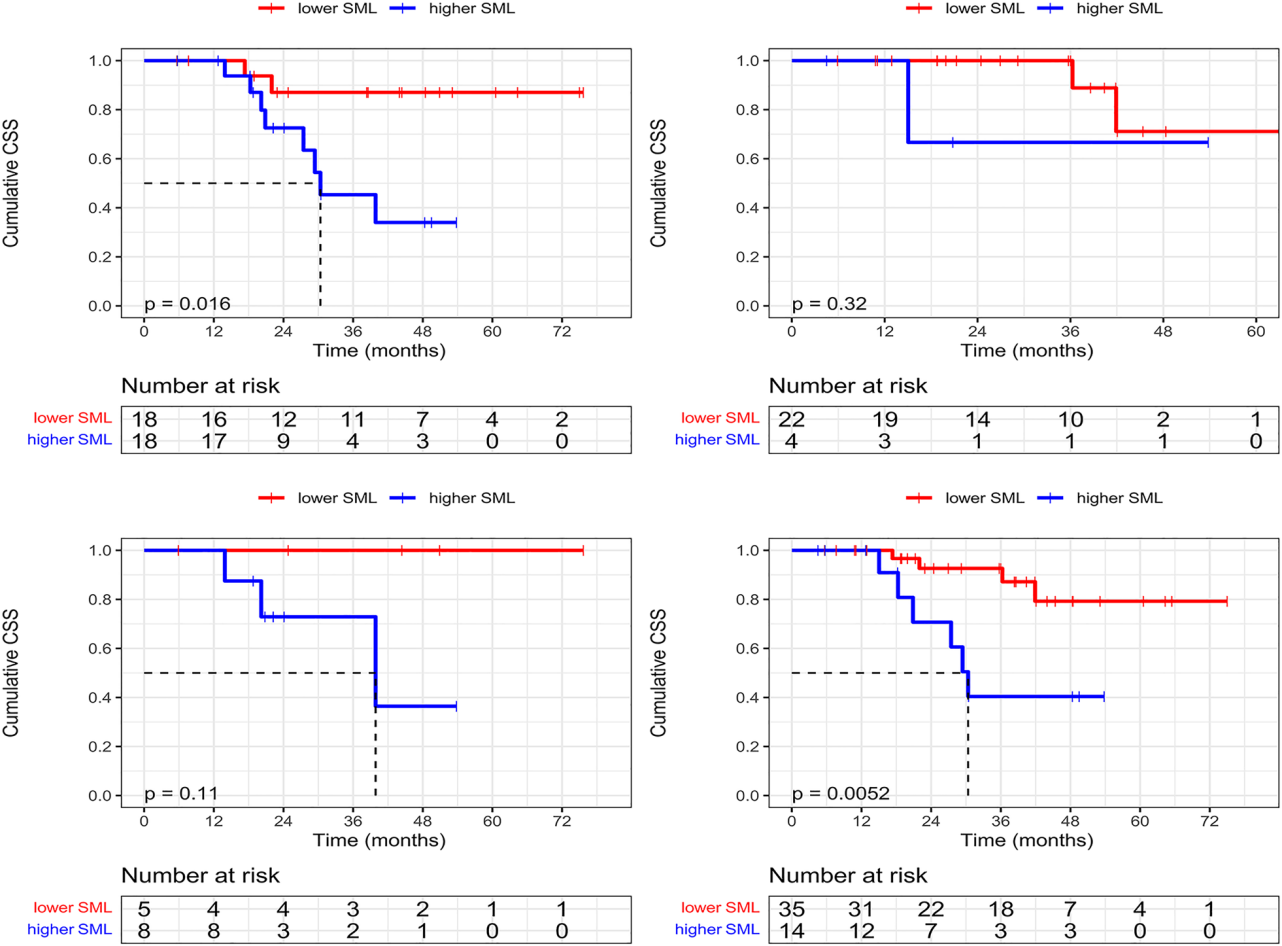
Subgroup analyses for CSS between lower and higher SML groups. **a** Patients with higher SML had a reduced CSS in the patients with cirrhosis. **b** No significant difference was found in CSS between patients with lower and higher SML subgroups in non-cirrhosis patients. **c** Trend towards an impaired CSS was observed in patients with higher SML in patients with MVI. **d** Patients with higher SML had a reduced CSS in the patients with non-MVI

Subgroups analyses were performed as per imbalanced variables between lower and higher SML groups, thereby minimizing the effects of confounding factors. As exploratory analyses showed, the higher SML was associated with reduced CSS in all subgroups stratified by the presence of cirrhosis or MVI (cirrhosis subgroup: *P* = 0.016, Fig. 6a; non-MVI subgroup: *P* = 0.005, Fig. 6d), despite with an equivocal difference in the non-cirrhosis subgroup and MVI subgroup ((*P* = 0.319, Fig. 6b; *P* = 0.110, Fig. 6c).

Multivariate analysis for CSS identified AFP > 400 ng/mL (HR:5.643, 95%CI:3.608–17.833, *P* < 0.001) and SML < − 2.42% (HR:6.586,95%CI:3.610-22.210, *P* < 0.001) were independently predictive of poor CSS (Table 2).

## Discussion

In this retrospective study, the skeletal muscle index of HCC patients experiencing curative hepatectomy plus adjuvant TACE significantly decreased over 6 months. More skeletal muscle mass loss served as an independent predictor of poor survival for HCC patients.

In advanced HCC patients, low SMI or sarcopenia was more common and proved to have a negative impact on these patients undergoing systemic therapy or intra-arterial therapy [17–19]. Indeed, the incidence of low SMI or sarcopenia in HCC patients who received curative treatments was also high and worthy of attention. In this study, low SMI patients accounted for 37.1% of study subjects, which was similar to 14.0–40.3% reported in previous studies [20–23]. Comparing to distribution of age or sex and the frequency of liver cirrhosis or chronic liver disease among these studies, it seems that the prevalence of low SMI was in accordance with liver disease progression. Besides, the effect of tumor burden led to reduced SMI due to decreasing appetite, increasing protein decomposition and aggravation of systemic inflammatory response [19, 24].

In addition, some prior studies also identified either low SMI or sarcopenia as indicative of recurrence and reduced survival for HCC patients undergoing hepatectomy [20, 25–27]. However, this study indicated that preoperative SMI did not stratify liver-related mortality risk, which could be a result of different endpoint and additional adjuvant TACE treatment.

When the presence of risk factors determined after curative hepatectomy for HCC patients, the combined therapy, namely, curative hepatectomy plus adjuvant TACE, is an appropriate therapy. However, there were no studies elucidating the impact of skeletal muscle loss on such patients previously. As an integrated course of treatment aiming to achieve curative effect, skeletal muscle mass depletion should be quantified during a period of combined treatment rather than hepatectomy alone. Hence, the potential impact of TACE on skeletal muscle mass was considered in our study.

Our data showed that the median SML over 6 months was − 1.6% in the cohort of patients with Child–Pugh A class. A study found that the annual change rate in skeletal muscle mass at the level of L3 in counterparts was − 1.3% [28]. This demonstrated that SML during hepatectomy plus adjuvant TACE was also significant. In a study concerning changes in body composition for HCC patients underwent hepatectomy, the psoas muscle index was measured at different timepoints after surgery and measurements failed to exceed preoperative level in the following 2 years [9]. It is surmised that the loss of skeletal muscle mass caused by hepatectomy could last for a quite long time. Notably, skeletal muscle loss varied individually in this study. This could be attributed to the heterogeneity of individual liver function reserve and adjuvant TACE treatment.

Increasing evidence has demonstrated that more skeletal muscle loss may be related to poor tumor characteristics, such as MVI, larger tumor size and worse liver function, but not the treatment itself. Our results showed more skeletal muscle mass loss during treatment was associated with cirrhosis, MVI and inflammatory status, which is in line with the findings of other studies, and a negative correlation was observed between SML and liver cirrhosis as well as MVI. Recently, researchers have found that there was a distinct decrease in skeletal muscle mass per TACE cycle, and the degree of skeletal muscle mass depletion was a risk factor for overall survival [10, 11]. They further proposed that the SML after the first TACE largely reflected worse tumor characteristics, and the liver function reserve was the main factor affecting SML in subsequent TACE cycles. Similarly, a study enrolling HCC patients who underwent radiotherapy to the liver demonstrated that HCC patients newly diagnosed with sarcopenia after radiotherapy had more Child–Pugh B or C as well as a larger tumor burden [29]. Another report including 603 patients with cirrhosis identified a higher NLR level in patients with severe sarcopenia than those in non- and sarcopenic groups [30]. In addition, Voron, T. et al. [20] thought sarcopenia was associated with HCC satellite lesions and MVI.

In the light of metabolic syndrome and obesity representative of muscle–liver–adipose tissue axis disorder, which has been underlined in the progression of skeletal muscle depletion, BMI was used for further stratification of SML subgroups. In the higher SML group, the subgroup analysis demonstrated that obesity patients had a trend of declining survival rate compared with non-obesity patients. In the study by Yabusaki, N. et al. [25] showed, HCC recurrence rate in patients with BMI ≥ 22 was significantly different between patients with or without sarcopenia after hepatic resection. Accordingly, sarcopenic obesity was regarded as a special categorization in the sarcopenic patients, and consisted of skeletal muscle loss and accumulated adipose tissue, probably related to worse performance status and poor prognosis in HCC patients.

This study revealed that SML could evidently differentiate survival stratification. In spite of no statistical significance in some subgroups, it is probably related to the limited sample size. In the comparison of SMI, skeletal muscle mass loss and its rate may be more likely to reflect the response of patient’s condition to therapeutic intervention. A cohort study also showed that the rate of muscle loss was a robust predictor for mortality in patients with cirrhosis instead of other single muscle mass-related measurements [31].

Our results showed that SML < − 2.42% remained a significant independent risk factor for reduced CSS, indicating that the effect of skeletal muscle loss on prognosis was not the result of concomitant presence of adverse factors but rather the progression of liver dysfunction and worse general clinical condition. A mounting number of studies have unveiled that skeletal muscle mass loss was associated with pathophysiological alternations in the body, including decreased hepatic glycogen synthesis, hyperammonemia, glycogenolysis, myostatin, autophagy, and proinflammatory cytokines as well as endocrine changes [1, 32, 33]. However, more explorations are required in the underlying mechanisms.

To date, there is only general guidance for the prevention and treatment of sarcopenia, without a distinct role on decision-making in clinical settings. Our study suggested individual skeletal muscle loss and its rate were quite remarkable, especially for HCC patients with cirrhosis. Accordingly, it is necessary to increase awareness of declining skeletal muscle index during radiologic follow-up. If obvious skeletal muscle loss is observed, nutrition-supporting interventions, such as late evening snack, branched-chain amino acid supplementation and in-hospital exercise, should be considered [34–36].

The limitation of this study should be recognized. First, the data were retrospectively collected and analysed from a single center. Second, due to a lack of skeletal muscle function evaluation, sarcopenia could not be defined strictly, but the loss of skeletal muscle mass could be precisely quantified[37]. Third, because of the limited sample size of certain subgroup, some conclusions need to be further validated.

## Conclusions

In conclusion, skeletal muscle loss during curative hepatectomy and adjuvant therapy has a detrimental impact on liver-related survival for patients with HCC, and seems to indicate poor liver and tumor characteristics.

## Data Availability

The data sets generated or analyzed during the study are not publicly available due to the data of DICOM including patients’ intimate information but are available from the corresponding author on reasonable request.

## Appendix

See Table 3.

## Abbreviations

SMI: Skeletal muscle index
SML: Skeletal muscle loss
HCC: Hepatocellular carcinoma
CLD: Chronic liver disease
TACE: Transarterial chemoembolization
CSS: Cause-specific survival
CT: Computed tomography
MVI: Microvascular invasion
BCLC: Barcelona Clinic Liver Cancer Group
ROC: Receiver operating characteristic
NLR: Neutrophil to lymphocyte ratio
L3: The third lumbar vertebra level
